# Use of an Unguided, Web-Based Distress Self-Management Program After Breast Cancer Diagnosis: Sub-Analysis of CaringGuidance Pilot Study

**DOI:** 10.2196/19734

**Published:** 2020-07-06

**Authors:** Robin M Lally, Kevin Kupzyk, Steve Gallo, Donna Berry

**Affiliations:** 1 College of Nursing Fred & Pamela Buffett Cancer Center University of Nebraska Medical Center Omaha, NE United States; 2 College of Nursing University of Nebraska Medical Center Omaha, NE United States; 3 Center for Computational Research Roswell Park Cancer Center Buffalo, NY United States; 4 School of Nursing University of Washington Seattle, WA United States

**Keywords:** eHealth, psychoeducation, supportive oncology, distress, self-management, oncology, breast cancer

## Abstract

**Background:**

Unguided, web-based psychoeducational interventions are gaining interest as a way to reach patients while reducing pressure on clinical resources. However, there has been little research on how patients with cancer use these interventions.

**Objective:**

The objective of this analysis was to evaluate how women newly diagnosed with breast cancer used the unguided web-based, psychoeducational distress self-management program CaringGuidance After Breast Cancer Diagnosis while enrolled in a pilot feasibility study.

**Methods:**

Women with stage 0 to II breast cancer diagnosed within the prior three months were recruited from clinics primarily in the Northeastern United States for participation in a 12-week pilot study of CaringGuidance plus usual care versus usual care alone. Usage prompts included sets of emails sent weekly for 12 weeks; standardized congratulatory emails after every two hours of program use, and informative emails for each cognitive-behavioral exercise. Individual user activity on the site was automatically tracked by an analytics system and recorded directly in the CaringGuidance database.

**Results:**

Complete usage data were available for 54 subjects. Ninety-eight percent of the intervention group logged into CaringGuidance independently at least once. Thirty-eight (70%) logged in during all three months, 15 (28%) were intermittent users, and one (2%) was a non-user. Users (n=53) averaged 15.6 (SD 9.85) logins. Mean logins were greatest in month 1 (7.26, SD 4.02) and declined in months 2 (4.32, SD 3.66) and month 3 (4.02, SD 3.82). Eleven (21%) used CaringGuidance with both the frequency and activity level intended at study outset, 9 (17%) exceeded intended frequency and activity (high-high users), and 10 (19%) were below expected usage on both login frequency and activity (low-low users). Low-low users and high-high users differed significantly (*P*<.001) in the total number of views and unique views of all program components. Change in depressive symptoms and the number of sessions (r=.351) and logins (r=.348) between study months 1 and 2 were significantly correlated (*P*=.018, .019). Higher baseline distress was associated with more unique views of program resources (r=.281, *P*=.043). Change in intrusive/avoidant thoughts from baseline to month 3, and the number of users’ unique exercise views were negatively correlated (r=–.319, *P*=.035) so that more unique exercise views, equated with greater decline in intrusive/avoidant thoughts from baseline to month 3.

**Conclusions:**

These findings favor the hypothesis that the key ingredient is not the amount of program use, but each user’s self-selected activity within the program. More research is needed on the ideal ways to maintain use, and capture and define engagement and enactment of behaviors by people with cancer accessing unguided, self-management web-based programs.

## Introduction

### Background

Interest in unguided, web-based psychoeducational and behavioral interventions is growing due to ease of delivery and need for few clinical resources. The outcomes of such interventions, however, rely on patients accessing and using these programs independently [1]. Identifying how populations of patients use web-based programs and the best way to support individuals in meaningful program use and engagement is an emerging science [2]. Perceived program quality and usability [3] and supporting potential users’ intent to and persistence in program use, are crucial to reducing use-attrition and increasing the likelihood that patients receive health benefits [4]. Duration of use may also be insufficient to describe the dose of web-based intervention necessary to achieve the intended benefit due to the inability to monitor off-line processing of program content, difficulty capturing the depth of online engagement [5,6], and not all users need to use the full program to attain their personal goals [7,8].

Moment-to-moment use of a website is one part of program users’ engagement and has been coined, “microlevel engagement” [6]. Together with measures of the depth of user interaction with the program, which results in the enactment of behavior change (ie, macrolevel engagement), micro- and macro-level engagement result in “effective engagement” [6]. At least some amount of program use, measurable through the program’s analytic system, must occur for there to be engagement and intervention effect. Analyses presented are focused upon the moment-to-moment use of a new unguided, web-based program for women with breast cancer.

Published reports on completely unguided, web-based psychosocial distress self-management interventions for adults with cancer are limited [9-13]. To the best of our knowledge, CaringGuidance After Breast Cancer Diagnosis [13-16] is one of only two such interventions specifically designed to address the psychosocial needs of women with breast cancer. The primary differences between CaringGuidance and the other program (BREATHE) [11] are that CaringGuidance is presented in English; was developed with input from, and includes vignettes of, Black as well as White American breast cancer survivors [16]; and is intended for initial use by women as soon as possible after diagnosis as opposed to after treatment has ended. While it is best to initiate cancer-related distress reduction to alleviate anxiety and depressive symptoms early after diagnosis [17], the initial post-diagnosis period is also busy with pre-treatment examinations, physician appointments, surgery, and chemotherapy. Therefore, it was expected that program use statistics for CaringGuidance might differ from unguided programs in which users access the program when treatment has been ongoing or is complete.

### Objective

The objective of this analysis was to evaluate how women newly diagnosed with breast cancer used the unguided web-based, psychoeducational distress self-management program CaringGuidance while enrolled in its first pilot study [13-15]. The overall goal is to further inform the science of unguided web-based interventions by describing the (1) frequency, duration, and activity of CaringGuidance use by women newly diagnosed with breast cancer in total and by the month of study participation; (2) baseline characteristics of women who demonstrated high, moderate, low, and no program use; and (3) to evaluate high, moderate, and low program users’ study completion, program satisfaction, enactment of distress management skills, and distress outcomes.

## Methods

### Participants

Women with stage 0 to II breast cancer diagnosed within the prior three months were recruited from clinics primarily in the Northeastern United States for participation in a 12-week pilot feasibility study of CaringGuidance plus usual care versus usual care alone. Enrollment was limited to stage 0–II breast cancer to reduce variation in the treatment experience among subjects. Details of recruitment and eligibility for this study of 100 women have been previously reported [13,14]. Institutional Review Board approval was received, and written consent obtained from all participants before study assessments. This feasibility pilot study, not involving drugs or devices, was not deemed eligible in 2013 for clinical trial registration by the university research administration.

### Intervention

Baseline demographic and psychosocial measures of distress (ie, Distress Thermometer, Center for Epidemiologic Studies Depression Scale, and Impact of Events Scale), as well as Social Constraints Scale on spouse/partner and family/friends, were completed following written consent. These measures were repeated at months 1-3. Participants randomized to the CaringGuidance user condition received individual usernames and passwords. Usernames and passwords cannot be changed by users, thus permitting tracking use by assigned usernames.

CaringGuidance After Breast Cancer Diagnosis (version 1) is an unguided, web-based, psychoeducational program based on theories of stress and coping [18], and adjustment to illness through cognitive processing of life-threatening events [19-22]. The program’s topical outline was guided by findings of the PI’s grounded theory describing women’s thought processes following diagnosis [23,24]. Interventional components of CaringGuidance are based on cognitive-behavioral, problem-solving, and supportive oncology techniques, which have demonstrated efficacy in both in-person and web-based delivery for the breast cancer population [25,26]. From 2011-2013, the program was developed in an iterative process of review and revision by a team of oncology professionals, including psychologists, breast cancer survivors, web developers/programmers, and software engineers [16].

CaringGuidance program components include 5 learning modules divided into 17 topical sections (Textbox 1). Resources include 90 video vignettes filmed with 6 breast cancer survivors (ages 30-70, stage 0-III breast cancer, equal representation of Black and White American women) and 20 self-management “cognitive-behavioral homework” exercises (eg, visualization, cognitive reframing). There are also 13 resource modules consisting of a glossary, links to the program exercises, a library of full-length videos from which the vignettes were derived, breast cancer risk factors, signs of depression and anxiety, links to cancer resources, and myths and facts about breast cancer. The five modules are listed in tabs at the top of each page. Pages include a list of topical sections within each module to provide quick access (Multimedia Appendix 1). Users can orient and navigate using breadcrumb navigation at the top of each page and the titles of the next and previous sections appearing at the bottom of each page. Additional descriptions of CaringGuidance may be found elsewhere [14,15].

At login, first-time users are directed by the program through three introductory pages, including a welcome video from the program creator/PI, instructions for use, and a 12-statement tailoring exercise that guides users to program modules based on their self-selected greatest concern. The purpose of tailoring in this program is to help match the content to each user’s needs with the expectation that this will increase content relevance [27] and contribute to users achieving their health goals [28]. Upon subsequent logins, users are directed to a personalized homepage but may access the introduction and tailoring exercise at any time. The 140 program components may be accessed by users at will in a flexible manner to direct their distress self-management. There is no required order in which to use the components nor requirements for completion before moving on to a different component.

Study participants were instructed that the suggested usage frequency and duration were 20-30 minutes for 2 to 3 times per week (range 40-90 minutes/week) for 12 weeks (480-1080 total minutes, 8-18 hours), but that they were permitted to access the program as much or as little as desired to create their own experience. The frequency/duration suggested was based on the traditional 12-week program of 1-hour counseling sessions. It was not expected that all users would access all components as everything within the program was not relevant to every user. Participants were informed during the consent process that program use would be tracked by the program’s analytic system in a manner invisible to them.

At enrollment, research staff showed participants the login page and the first page containing the welcome video but did not go further into the program. When enrollment was completed by email, an explanation of the first three program pages was provided in the email containing the user’s login information. All participants received a hard copy pictorial guide on basic website use (eg, how to enlarge the font and adjust the volume).

During the study, usage prompts included standard emails sent weekly for 12 weeks supporting continued use or encouraging use; standard congratulatory emails after every two hours of program use, and pre-written informative emails for each cognitive-behavioral exercise sent following two or more minutes of a user accessing that component (these emails also directed users to relevant program components for self-reflection). All emails were automated or sent by staff using a CaringGuidance Gmail account to simulate an automated message, thus avoiding personal interaction. To assess safety and review of the daily symptom/support log maintained by all participants, one research assistant phoned monthly for a scripted conversation. These calls averaged 18.8 minutes (SD 7.73) in month 1, down to a mean 13.5 minutes (SD 4.64) in month 3. The script directed the research assistant to suggest portions of the program not yet accessed during the call. Calls were recorded and 10% reviewed by the principal investigator to assure script adherence. Emails and calls placed by participants to the research office for technical support (n=6) were tracked [14].

**CaringGuidance modules and topical sections**

- Are my reactions normal?
  Fears and anger
  Exploring other emotions
  Why might I think about cancer differently than other women?
- What does this diagnosis mean?
  Why me? or Why not me?
  Questions and misconceptions
- Who am I now?
  Self-concept
  Accepting support
  Is a support group right for me?
  The meaning of survivor
  Body image and sexuality
  How will people act toward me now?
- What are strategies to care for myself?
  Coping with cancer
  Talking with people around you
  Personal control strategies
- Moving forward
  Personal growth from this experience
  How much will cancer be a part of my life?
  Setting healthy goals

### Usage Data Storage and Retrieval

The CaringGuidance web analytics system was developed by our team to collect user activity information that would facilitate the analysis of various usage modalities. The activity of individual users on the site was automatically tracked by the system and recorded directly in the CaringGuidance database. Each time a user visited an individual page on the site (referred to as a “page visit”), the analytics system collected the information specified in Textbox 2.

**Information collected by the analytics system.**

- The name and internal identifier of the user,
- Whether or not the activity is a login event (eg, the user logged into the site using their username and password),
- A session identifier that facilitates the tracking of a user’s activity during a particular visit,
- Whether or not the user viewed a resource, article, exercise, or video and which of these items were viewed
- A timestamp with second resolution

### Usage Definitions and Measures

**Figure 1:**
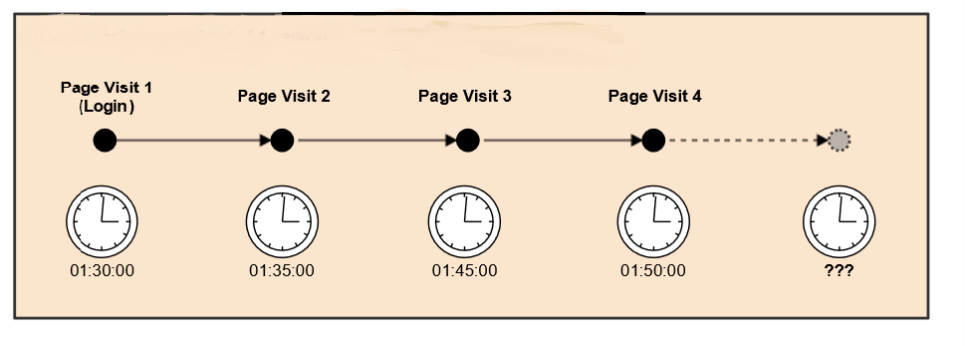
User session comprised of 4-page visits where clocks represent page view timestamps. The user visited for at least 20 minutes; however total time is underestimated as it is not known how long the user spent on the site during page visit 4.

**Figure 2:**
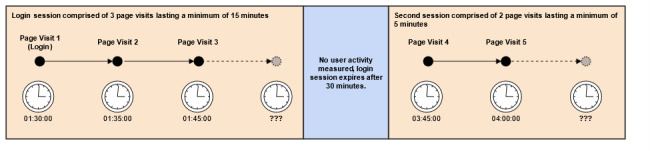
User session wherein a period of inactivity triggers use to be treated as two individual sessions.

Page visits and login information stored in the database were used to construct a set of sessions that provided details as to how each user utilized the site during that time. We defined a session as a set of consecutive page visits, starting with a login event and ending with a logout or a period of inactivity. The amount of time that a user spent on any given page visit was calculated using the difference between the timestamps of the current page visit and the next page visit within the session. Because users may simply close their web browser, turn off their computer, or leave their computer for some time in addition to clicking the “logout” button, we did not have a reliable method for calculating the time spent on the final page visit in a session (Figure 1). To overcome this limitation, we used a configurable period of inactivity to infer a logout event and expired the session—currently set to 30 minutes. In the case where a user’s session expired, and they later returned to the site, this activity was treated as a new login event and initiated a new session for that user (Figure 2). The activity/inactivity rules provided a lower bound for the time that a user visited CaringGuidance during any given session.

#### Usage

In this current micro-level engagement analysis, usage was defined as frequency, duration, and user program activity. This definition is consistent with definitions of usage applied in prior research [29]. The *frequency* was defined as the number of logins to CaringGuidance per participant per study month over the 3-month study enrollment. Frequency also was characterized by the number of sessions in which users engaged per month and over the study enrollment period, since more than one session may have been engaged in during a single login (Figure 2). The *duration* was defined as the total number of minutes logged in each month and over the 3-month study period. The *activity* was defined as the number of total page views and the number of unique page views per participant per month and overall for each of the CaringGuidance program components (ie, module, exercise, videos, and resource pages). Finally, activity was examined for which components appeared to be preferred based on use and repeated access.

#### Users and Non-Users

It was expected that participants would access CaringGuidance with varying frequency and duration for multiple reasons, including the recency of their cancer diagnosis, levels of distress, and ongoing treatment. *Non-users* were defined as study participants who received a username and password but who registered ≤1 log-ins/sessions and for whom zero minutes were recorded. Consistent with definitions described by van den Berg et al [29], *continuous users* were defined as registering at least one login/session per month for each of the 3 study months. *Intermittent users* were defined as those who logged in initially and registered at least one login/session in study month 1 but then registered <1 session in study month 2 or 3, or both study months 2 and 3.

#### Macrolevel Engagement

For this pilot study, macro-level engagement [6] was measured with three questions rated from 1 (strongly disagree) to 5 (strongly agree) on the self-report satisfaction survey at the conclusion of month 3 [14]. These questions were, “I used things I learned from CaringGuidance to change (a) my thoughts about breast cancer; (b) how I talked or acted around people; and (c) my self-care behaviors.

#### Participant Usage Groups

In order to characterize CaringGuidance usage concerning baseline and monthly psychological outcomes, user patterns were divided into three usage groups designated as low, moderate, and high. In so doing, we were inspired by definitions used by van den Berg et al [29]. For our usage analysis, *low frequency* was defined as 1-12 logins total throughout the 12 weeks; in other words, 50% or less of the minimum number of logins suggested to participants (ie, 24 logins) at enrollment. *Moderate frequency* was defined as 13-24 logins throughout the study, and *high frequency* was defined as ≥25 logins over the 12 weeks. Activity was likewise defined as *low activity* equal to opening 0-25% of the program’s 140 components (ie, unique views), *moderate activity* equal to opening 26-50%, and *high activity* equal to opening 51-100% of the program components. High activity was defined as viewing >50% of the components because not all components applied to all study participants. Logins and activity were cross-tabulated to characterize use as *low-low*, *moderate-moderate*, or *high-high*.

Total duration logged into the program was not used in the usage group calculation because of varying speeds of Internet connections, differing participants’ reading speed, differing times needed to use various program components, and the analytics systems’ inability to measure the duration of the last session, thus underestimating total time on the program.

## Results

### Demographics

Study participants were women diagnosed with stage 0 to II breast cancer within the prior three months at baseline. Ability to read English and access a computer with Internet service and email were required since the program at that time was not mobile accessible. Fifty-seven participants were randomized to the CaringGuidance condition; however, one participant did not receive a password until week 5, and two participants withdrew after randomization due to feeling too busy to participate. Thus, CaringGuidance analytic data were available for 54 participants.

These 54 participants ranged in age from 36 to 78 years (mean 55.02, SD 9.4). They reported prior experience using the Internet, with a median of 1-hour Internet use (range 10 to 600 minutes) per day at baseline. Of these 54 participants, the majority were White (n=50, 93%) and married/partnered (n=32, 59.3%), while 22 (40.7%) were single/divorced/widowed. Forty-nine of the 54 (91%) had attended at least some college. Most were employed full-time (n=33, 61.1%) while 10 (18.5%) reported part-time employment at baseline, and of those reporting income (n=47), 53.2% had a household annual income of $75,000 or above. See Textbox 3 for clinical characteristics. Full demographic data on the 100 participants enrolled in the pilot study have been previously reported [13].

**Clinical characteristics and treatment during the study (N=54).**

- Cancer stage at baseline
  0 (n=13)
  I (n=24)
  II (n=16)
  “early” (n=1)
- Time since diagnosis at baseline
  <4 weeks (n=20)
  1-2 months (n=28)
  2-3 months (n=6)
- Breast surgery procedure during the study (n=29)
- Chemotherapy received during the study (n=33)
- Radiation therapy received during the study (n=43)

### Attrition

Eight participants assigned to the CaringGuidance condition withdrew or were lost to follow up, meaning that they did not complete all study psychosocial measures [14]. These include the 3 participants noted above who did not receive their login information or withdrew after randomization. The other 5 participants continued to use CaringGuidance despite not completing all psychosocial measures. Four of these were intermittent users (ie, logged in only in month 1) while one was a continuous user (ie, logged in in all three study months). These five participants did not differ demographically at baseline from others assigned to the CaringGuidance condition.

Of the 54 CaringGuidance participants for whom usage data are available, 38 (70%) were continuous users, 15 (28%) were intermittent users, and one (2%) was a non-user. The non-user was age 62, Black, unemployed and widowed, with Stage 0 breast cancer. She completed the psychosocial surveys and monthly calls with the research assistant, during which she indicated her intention to use a library computer to access CaringGuidance, but transportation barriers prevented her from doing so. The “non-user” was eliminated from this analysis and findings for the 53 “users” (continuous or intermittent) with mean age 54.9 years (SD 9.4), and 94% White are reported here.

### Frequency

**Figure 3:**
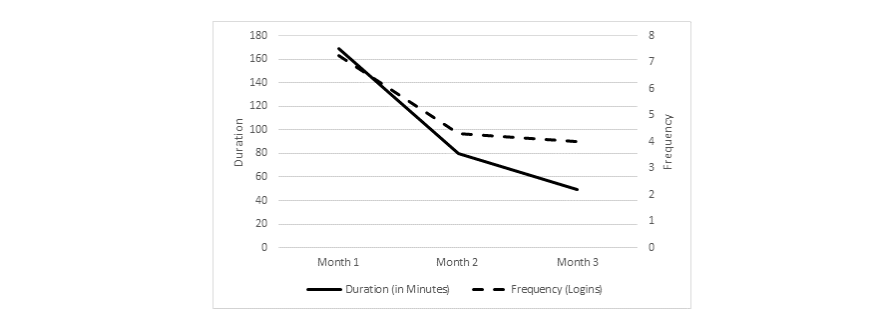
Change in login frequency and duration of program use over 3 months.

Users (n=53) logged in an average of 15.6 (SD 9.85) times during their 12-week access period. The mean number of logins was highest in month 1 (mean 7.26; SD 4.02) and declined after that such that the mean logins in month 2 were 4.32 (SD 3.66) and 4.02 (SD 3.82) in month 3. Overall, login attrition was significant across all three study months (*F*_2,104_=28.9, *P*<.001) with the sharpest decline in logins occurring between study months 1 and 2 (*F*_1,52_=38.8, *P*<.001) (Figure 3).

Users averaged 16.94 sessions (SD 10.42) over 12 weeks. Session attrition mimicked login attrition, as would be expected, with the most considerable decline in sessions between months 1 (mean 7.85, SD 4.34) and 2 (mean 4.83, SD 4.28; *F*_1,52_=27.4, *P*<.001) and remaining stable from month 2 to month 3 (mean 4.26, SD 3.84; *F*_1,52_=17.0, *P*=.217).

With one exception, no significant correlations were identified between use frequency (number of logins or sessions) and change in psychological outcomes; the one exception being a significant positive correlation between change in depressive-symptoms and the number of sessions (r= .351) and logins (r= .348) between study months 1 and 2 (*P*=.018 and .019 respectively), but not between any other study months.

### Duration

Time spent on CaringGuidance by users during their 12 weeks of access ranged from 9.27 to 1265.3 minutes (21.1 hours; mean 4.98 hours, SD 3.61). The mean duration of use declined significantly over the 3 study months from 169.38 minutes (SD 120.08) in month 1 to 49.06 minutes (SD 45.06) in month 3 (*F*_2,104_=40.2, *P*<.001). The decline in duration of use was also significant when examined by month; from month 1 to month 2 (F_1,52_=33.7, *P*<.001) and month 2 to month 3 (F_1,52_=7.9, *P*=.007).

The total duration of CaringGuidance use was found to have a significant positive relationship (r=.291, *P*=.036) with users’ baseline intrusive/avoidant thinking such that users with more initial intrusive/avoidant thinking used CaringGuidance for more total minutes over the 3 study months [14,15]. This was also true for baseline spouse/partner derived social constraints and the total duration of CaringGuidance use (r=.370, *P*=.031) and minutes of use in study month 2 (r=.422, *P*=.013) and month 3 (r=.345, *P*=.045) [15]. No additional statistically significant correlations were found between duration and baseline or month 3 psychological outcomes.

### Activity

#### Modules

The 17 written learning component pages (modules) were accessed 1 to 103 times per user (mean 41.11, SD 26.57). All 53 users accessed at least one module. There were between 1 and 17 *unique* module page views per user (mean 12.06, SD 4.65), meaning that all module pages were accessed at least once. Eleven users (21%) accessed all 17 module pages. The five most viewed modules were: “Fears and Anger” (211 views), “Why me? Why not me?” (218 views), “Self-concept” (242 views), “Personal growth from this experience” (243 views), and “Coping with cancer” (290 views). Least accessed modules were: “Is a support group right for me?” (51 views), and “How will people act toward me now?” (59 views).

#### Exercises

Each of the 20 cognitive-behavioral exercises was accessed from 33 to 199 times [14]. Fifty-two of the 53 users (98%) viewed exercises yielding between 0 and 162 exercise views per user (mean 38.91, SD 34.69). Of these, there were between 0 and 20 *unique* exercise views (mean 12.06, SD 5.61), meaning that some users viewed the same exercises multiple times. Repeat viewing was encouraged by several exercises that directed users to review prior exercises as a means to self-monitor changes in thinking over time. Six of 53 users (11%) viewed all 20 exercises.

#### Videos

Fifty users (94%) accessed videos resulting in between 0 and 119 video views per user (mean 29.7, SD 28.29). Unique video views ranged from 0-80 (mean 23.91, SD 21.17). Eighty-nine of the 90 videos (99%) were viewed. The 10 most viewed videos were viewed between 29 to 42 times and featured four of the six survivors (two Black and two White American survivors). An overall theme of these most viewed videos was self-concept as a newly diagnosed survivor.

#### Resources

Each of the 13 resource components was accessed from 30 to 252 times by program users. Fifty-one of 53 users (96%) accessed at least one resource page. Three users (6%) accessed all resource pages. Resource pages were accessed between 0 and 67 times per user (mean 20.91, SD 14.93). On average, 7.62 (SD 3.62) of the resource page views were *unique*, indicating that some users returned to the same resource pages multiple times to review the content. The five most accessed pages were: breast cancer risk factors (96 times), cancer information resources (98 times), common questions about breast cancer (99 times), mindfulness-based stress reduction (109 times) and all program exercises list (252 times). Least accessed were questions to ask your doctor (30 times) and first appointments—talking with your doctors (40 times).

**Figure 4:**
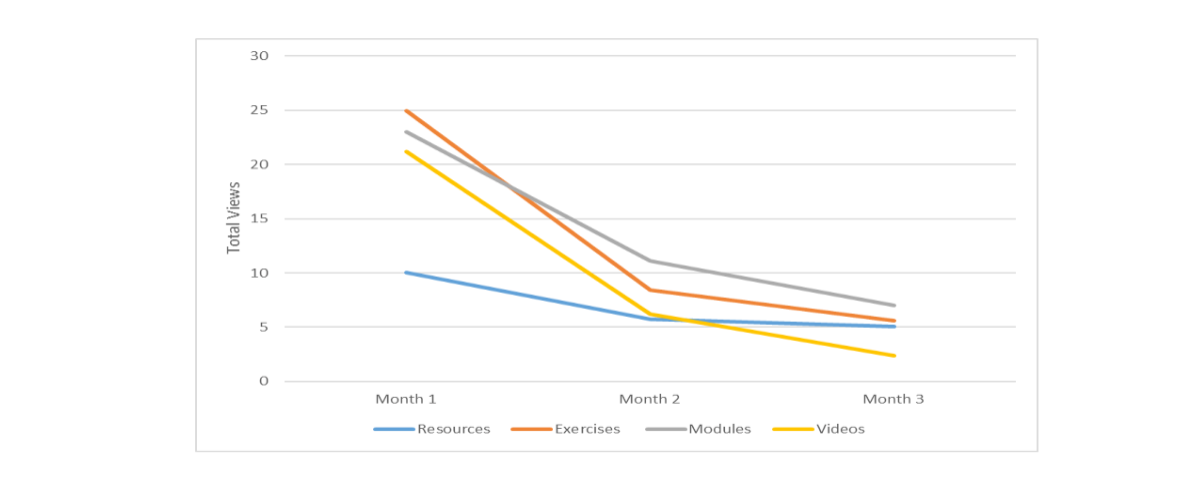
Program component views over 3 study months.

Overall, the most significant program activity occurred in month 1 for all components and significantly decreased between months 1 and 2 (*P*<.001). Views of program modules and videos continued to decrease between study months 2 and 3 (*P*=.004 and .003) (Figure 4).

### Psychosocial Associations

Baseline overall distress correlated positively with activity such that higher baseline distress was associated with more unique views of program resources by users (r=.281, *P*=.043). Additionally, at study month 3, the number of unique module views was negatively associated with spouse/partner derived social constraints (r=–.418, *P*=.021). In other words, more unique modules were viewed by subjects when perceived social constraints were lower.

When the *change* in overall distress, depressive-symptoms, and intrusive/avoidant thinking was examined, no correlations were found with user activity, with one exception. A significant negative correlation (r=–.319, *P*=.035) was identified between the change in intrusive/avoidant thoughts from baseline to month 3 and the number of users’ unique exercise views. In other words, the higher number of unique exercise views, the more significant the decline in intrusive/avoidant thoughts from baseline to month 3.

### Use Groups

Eleven of 53 (21%) users were defined as moderate program users. In other words, they used CaringGuidance in the range that approached the intended usage told to them at study entry for *both* frequency and activity (*mod-mod*). Nine users (17%) exceeded intended usage for *both* login frequency and activity (*high-high*), while 10 users (19%) were below expected usage on *both* login frequency and activity (*low-low*). In total, 39 (74%) users were in the moderate to high activity range during the period they were logged in the program, and 30 (57%) logged-in with moderate to high frequency. Overall frequency and activity were highly associated (r=.565, *P*<.001). Low-low users and high-high users differed significantly (*P*<.001) on the total number of views and unique views of all program components.

#### Low Frequency/Low Activity (Low-Low)

**Figure 5:**
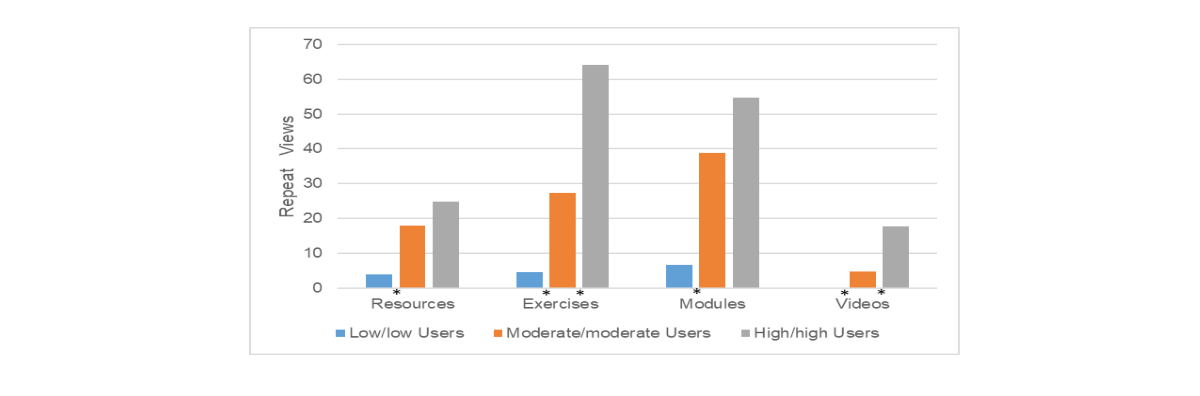
Repeat views by user group. An asterisk indicates significant differences between adjacent groups (left to right): Resources *P*=.002; Exercises *P*<.001 and .012; Modules *P*<.001; Videos *P*=.001 and .016.

Three of the 10 low-low users were also part of the group who did not complete any of the study’s monthly mailed psychological assessments, and one of these subjects formally withdrew due to feeling too busy to participate. The low-low use group’s number of logins ranged from 2 to 10 (mean 5.2, SD 3.26). This group consisted of the one subject of all 53 users who viewed no exercises, the two subjects who viewed no resources, and the three subjects who viewed no videos. Low/low user activity involved 0 to 13 unique exercise views per subject (mean 5.3, SD 3.62) of the possible 20, 1 to 14 unique module views per subject of a possible 17 (mean 5.9, SD 3.87), 0 to 10 unique views of resource pages per subject of a possible 13 (mean 3.7, SD 3.62), and 0-18 unique video views per subject of a possible 90 (mean 4.0, SD 5.44). Low-low users returned to view individual study components, on average, 3.8 (resources) to 6.6 (modules) times, exclusive of videos. No videos were viewed a second time by these users (Figure 5). Despite being low on frequency *and* activity, 30% of subjects in the low-low group were “continuous” users, logging-in 8 to 10 times each over the 12-week study.

#### High Frequency/High Activity (High-High)

In contrast to the low-low user group, the 9 high-high users returned all monthly psychosocial assessments and completed the study. All 9 were “continuous” users logging-in from 25 to 30 times (range) throughout the study (mean 27.3, SD 2.0). Their activity involved 15 to 20 unique exercise views per subject of a possible 20 (mean 18.56, SD 1.67; 33% accessed all 20 exercises), 10 to 17 unique module views per subject of a possible 17 (mean 15.56, SD 2.35; 56% accessed all of the modules), 8 to 13 unique views of resource pages out of a possible 13 (mean 10.56, SD 1.74; 1 subject accessed all resource pages), and 34 to 74 unique video views per subject of a possible 90 unique video views (mean 51.0, SD 14.76). High-high users returned to view program components an average of 17.7 (videos) to 64.1 (exercises) times. The most return views were to exercises and modules (mean 54.8 views). This behavior resulted in significant differences in repeat viewing behavior between usage groups (Figure 5).

#### Demographic Characteristics of User Groups

There were no significant differences identified in the demographic characteristics (age, race, education, employment status, income, breast cancer stage, pre/post-op status, and self-rated knowledge about breast cancer at baseline) of low, moderate, and high login frequency users nor users with varying amounts of activity. Additionally, no significant difference was found between pre-study self-reported daily Internet minutes across low, moderate, or high CaringGuidance login frequency or activity. This remained true when low-low and high-high groups’ demographic characteristics were compared.

### Psychosocial Outcomes by User Group

Study outcomes on psychological distress indicated that access to CaringGuidance versus not was favorable for the intervention group as evidenced by significant differences in the reduction of distress and depressive symptoms between study months 2 and 3 [13]. When low, moderate, and high number of logins and low, moderate and high activity groups were examined individually, or combined low-low, mod-mod, or high-high, no significant differences in change for overall distress, depressive-symptoms, intrusive/avoidant thinking, or reports of social constraints from family/friends or spouse/partners were found from baseline to month 3 or between study months. In other words, no usage group was superior to another regarding the change in distress over the study period as represented by these variables.

### User Satisfaction Survey Completion

The CaringGuidance user satisfaction survey was completed by 60% (n=6) of the low-low users and 100% of high-high users. Low-low and high-high users did not differ significantly in CaringGuidance satisfaction except that the high-high users perceived that using CaringGuidance increased their knowledge about their breast cancer (mean 4.56, SD 0.73) versus mean 3.50 (SD 0.84) of possible 5 points among low-low users, (*P*=.049). Forty-nine percent of survey respondents reported that CaringGuidance use changed their thoughts about breast cancer, 44% indicated that program content led them to change self-care behavior, and 40% reported that what they learned from CaringGuidance changed how they talked or behaved with people [14].

## Discussion

### Principal Findings

This analysis explored the unguided use of a web-based psychoeducational distress self-management program by women recently diagnosed with breast cancer. We described the characteristics of users and non-users, the frequency and duration of use, users’ activity within the program, and outcomes associated with different use patterns.

Ninety-eight percent of the women who proceeded in the study logged into the CaringGuidance program independently at least once after randomization, a very positive result given reports of other studies in which rates of initial login were less than 50% [30]. Our data closely approximates that of similar unguided programs tested in a research environment where 90% of those assigned to the intervention logged in [29]. Those who volunteer for research are likely a motivated population and, of course, are aware that they have committed to study participation. On the other hand, the usage results from this study are notable because subjects not only voluntarily logged into a website on their own time, they did so after recently receiving likely the worst news of their lives—a cancer diagnosis, and while making hospital and clinics visits to undergo tests, surgery, chemotherapy and radiation treatments. Of course, use attrition was significant after month 1, but this is also typical of web-based interventions [30-33]. We did not collect information on users’ reasons for waning use, which could represent that some users achieved their goal for using the program sooner than others (e-attainers) [8]. Research continues to be needed to understand better how people recently diagnosed with cancer may be motivated to use and engage with unguided programs.

We also found that, consistent with prior research, total duration users spent on CaringGuidance did not correlate with psychological outcomes. Neither did the sheer number of logins nor the number of program components viewed correspond with distress as measured in this study. In other words, simply more use was not better. Researchers have argued that it is the depth of engagement with the program and the ability to glean what the user desires to support their needs that matters [5,6]. Evidence varies, however, among studies concerning the effect of higher amounts of intervention use on psychological outcomes, with some finding more logins and time spent reduces distress [34]. In contrast, other studies found the number of logins and duration not to affect depression or anxiety [35]. The variability in findings is likely related to the uniqueness of interventions’ targeted populations, behaviors targeted by the intervention, and how program use is defined, supporting the need to evaluate micro and macro-engagement for specific interventions [6].

Based on our findings, we hypothesize that given at least a minimal amount of program use, it is the content that viewers access and whether that content engages and satisfies the user that holds the most significant import to psychological outcomes. While this hypothesis requires additional testing, support is provided by our finding that the higher number of unique exercise views made by users, the more significant their decline in intrusive/avoidant thoughts. This finding supports the mechanism of action of the cognitive-behavioral influenced program exercises, which are intended to assist users to reframe their thinking and process the cancer experience, in turn reducing intrusive/avoidant thoughts over time.

Additionally, our findings demonstrated that women who reported higher depressive symptom scores logged in with greater frequency over months 1 to 2; in turn, significant differences between months 2 and 3 in depressive symptoms favored the intervention over control [13]. Although it is acknowledged that correlation does not inform us whether depressive symptoms led to more frequent logins or visa-versa, it is logical to presume that if logging in increased women’s feelings of depression, they would have stopped this voluntary activity.

Repeated views of program components favor the hypothesis that women found value in using CaringGuidance. If users found no value, they would not have returned dozens of times to multiple components, as we saw in this study. Similar conclusions have been drawn in prior studies [29]. Not only did we note repeated viewing of program components, but a greater propensity among high frequency/high activity users compared to other users to return to all components while showing repeat interest, particularly in program exercises and written content. This finding is promising because these components convey the program’s cognitive-behavioral ingredients, and thus, further development of these types of components is supported. Also promising is that the most accessed program modules focused on coping, personal growth from the cancer experience, and supporting survivors’ self-concept. The most viewed videos also dealt with self-concept, indicating that CaringGuidance users independently focused on program components that were meaningful for distress reduction through confronting and re-evaluating how cancer reshaped their identity and world view which are vital to cognitively processing the cancer experience [19-24].

Finally, we were surprised to find that there was no relationship between high distress and greater use of program videos. At the time of design, it was anticipated that women with more considerable distress at baseline would gravitate toward the program videos as an activity that took less focused attention. At the same time, those further in their ability to cognitively process the diagnosis would attend to program exercises. Breast cancer survivors involved in program development expressed concern over overwhelming distressed women, which in turn inspired our addition of more videos, the glossary of terms, and “Questions for your doctor” resource components. The hypothesis seemed to be somewhat supported in that women distressed at baseline gravitated to the program resources component, however women with intrusive/avoidant thoughts and experiencing spouse/partner social constraints used the overall program with greater duration and women with depressive symptoms used the program with greater frequency.

### Limitations

A limitation of this usage analysis was the potential underestimation of the total time users spent on the program because of the tracking system’s inability to define the time users spent on their last page visit of a session. This underestimation may be balanced somewhat by the fact that all participants in this pilot study received scripted monthly calls from the research assistant about which the primary purpose was to assess for adverse study events but included questions about users’ ability to login and find the information they sought in the program. Although it was not found that users viewed program components that were discussed during the calls, the contact may have prompted more program use than if users had only received the email prompts [36,37]. Other researchers have also found phone calls to be less effective than email prompts [38]. Data were not collected on whether email prompts were opened, although the sender (CaringGuidance program) likely acted as a weekly reminder. Additional limitations include the small, educated, and racially homogenous sample of women whom all had prior Internet experience and computer access, thus limiting generalizability. Lastly, although data were collected on user satisfaction and enactment of behaviors gleaned from the program, data were not systematically collected that would allow for analysis regarding reasons for use attrition.

### Conclusions

In conclusion, distressed women recently diagnosed with breast cancer self-selected the CaringGuidance program components that satisfied their needs and used these components with considerable variability in frequency, duration, and activity. These findings favor the hypothesis that the key ingredient is not the amount of use, but rather the self-selected activity of each user within the program. Given the ease of accessibility and low resource utilization associated with CaringGuidance as well as the safety, satisfaction, and preliminary efficacy findings [13-15], CaringGuidance offers a potential clinically implementable option for distress management in this population. Future work should focus on implementation such as encouraging women’s acceptance of mental health online support, helping users to find the components within the program that they desire, increase users’ motivation to continue using to their maximum benefit and to explore the depth of engagement and cognitive processing in which users engage off-line. Overall, more information is needed on the ideal ways to capture and define engagement and enactment of behaviors by people with cancer accessing unguided, self-management web-based programs.

## Appendix

Multimedia Appendix 1 Screenshot of pages from CaringGuidance™ After Breast Cancer Diagnosis Copyright © 2016 The Research Foundation for The State University of New York, licensed for academic research to the University of Nebraska.
