# Supplementary figures and images for: Use of an Unguided, Web-Based Distress Self-Management Program After Breast Cancer Diagnosis: Sub-Analysis of CaringGuidance Pilot Study
Source: J Med Internet Res. 2020 Jul 6;22(7):e19734. doi: 10.2196/19734 (PMC7381261; doi:10.2196/19734)

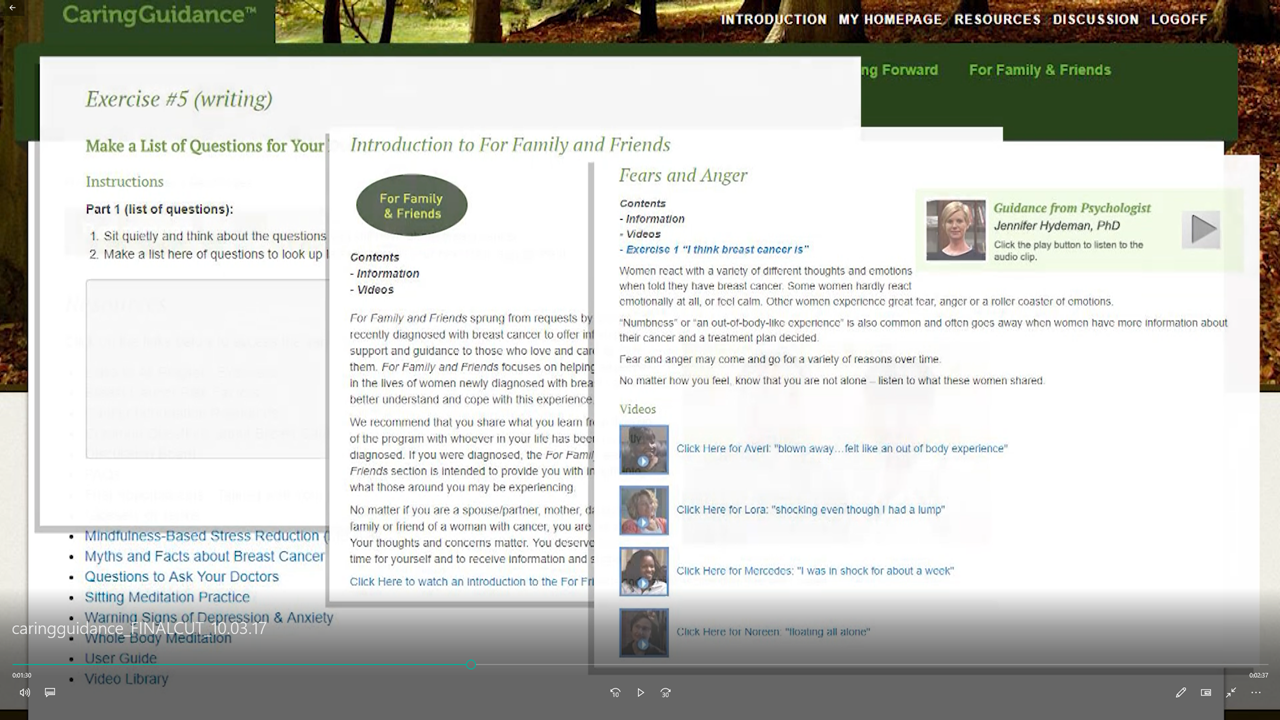

Supplement: Multimedia Appendix 1 [file jmir_v22i7e19734_app1.png]
